# STRETCHing HIV treatment: A replication study of task shifting in South Africa

**DOI:** 10.1371/journal.pone.0206677

**Published:** 2019-04-08

**Authors:** Baojiang Chen, Morshed Alam

**Affiliations:** 1 Department of Biostatistics and Data Science, University of Texas Health Science Center at Houston, School of Public Health in Austin, Austin, Texas, United States of America; 2 Department of Biostatistics, University of Nebraska Medical Center, Omaha, Nebraska, United States of America; Brigham and Women’s Hospital, UNITED STATES

## Abstract

The Streamlining Tasks & Roles to Expand Treatment and Care for HIV (STRETCH) program was developed to increase the reach of antiretroviral therapy (ART) for HIV/AIDS patients in Sub-Saharan Africa by training nurses to prescribe, initiate, and maintain ART. Fairall and colleagues conducted a cluster-randomized trial to determine the effects/impact of STRETCH on patient health outcomes in South Africa between 2008 and 2010. The purpose of our replication study is to evaluate Fairall and colleagues’ findings. We conducted push button and pure replication studies and measurement and estimation analyses (MEA). Our MEA validates the original findings: (1) overall, time to death did not differ between intervention (STRETCH) and control (ART) patients; (2) in a subgroup analysis of patients with CD4 counts of 201–350 cells per μL, the intervention group patients had a 30% lower risk of death than those in the control group, when controlling for baseline characteristics; (3) in a subgroup analysis of patients with CD4 counts of ≤200 cells per μL, time to death did not differ between the two groups; and (4) rates of viral suppression one year after enrollment did not differ between the intervention and control groups. This set of results have more caveats in the MEA. Although the intervention did not lead to improvements in the main outcomes, the effectiveness of STRETCH was proven to be similar to standard care while increasing the pool of prescribers, expanding their geographical range, and improving the quality of care for patients. Therefore, our analyses support the implementation of task shifting of antiretroviral therapy from doctors to trained nurses, which enhances confidence in the implementation of the intervention program and policymaking not only in South Africa but also in other developing countries that have similar circumstances.

## Introduction

The paper *Task shifting of antiretroviral treatment from doctors to primary-care nurses in South Africa (STRETCH)*: *a pragmatic parallel*, *cluster-randomised trial* by Fairall and colleagues [1] addresses a critical challenge to widespread treatment of HIV/AIDS in Sub-Saharan Africa. Although antiretroviral therapy (ART) regimes have proven efficacious in slowing the onset and symptoms of HIV/AIDS [2], dispensation of ART is hampered by the limited availability of doctors to prescribe the treatment and by the fact that doctors tend to be concentrated in urban areas [1]. In order to increase the reach of ART, the Streamlining Tasks and Roles to Expand Treatment and Care for HIV (STRETCH) program was designed to train nurses to prescribe ART (initiate and maintain on treatment) by introducing an educational outreach nurse training model [3–5]. However, information about the efficacy of the STRETCH program compared to the standard care system—in which only doctors can prescribe ART—is scarce [1].

Fairall and colleagues [1] conducted a cluster-randomized trial to determine the efficacy of STRETCH on patient health outcomes in South Africa between 2008 and 2010. Two cohort studies were conducted simultaneously to assess the effect of the intervention (STRETCH) compared to the standard care system when patients become eligible for ART initiation, and for individuals already enrolled in treatment programs [1]. Fairall and colleagues’ original hypothesis was that implementation of STRETCH would improve primary outcomes relative to standard care by expanding ART access. While this was not the case, they do note that STRETCH was not inferior to standard care. Additionally, the STRETCH program did improve several other health outcomes and quality of care indicators. Overall, no outcomes were worse in the STRETCH intervention groups than in the standard care groups [1]. Their findings provide support for expanding the pool of ART prescribers beyond doctors to nurses, thus increasing access to ART among populations not located near doctors, who are typically more widely available in urban settings.

Fairall and colleagues’ [1] study has been enormously influential in HIV/AIDS studies, leading to larger studies in this area and expanded application to other geographic locations [6–9]. Their findings reaffirm that task shifting of ART from doctors to trained nurses can benefit many HIV-positive patients in South Africa and other developing countries with similar circumstances, without negative impacts on key health outcomes and while improving their quality of care. STRETCH can also relieve doctors of a heavy patient burden and enable them to focus on more severely ill patients. This is essential in South Africa and other developing countries where shortages of doctors restrict access to ART. For example, studies in Rwanda, Cameroon and other Sub-Saharan African countries [6–9] have assessed the feasibility and effectiveness of task shifting from physicians to nurses due to shortage of physicians and other human resources for health, and reached similarly positive conclusions.

Our replication provides influential evidence for policymaking by supporting the results of prior studies. Validation of the findings can enhance confidence in the implementation of the intervention program and policymaking not only in South Africa, but also in other underserved areas with high burden of HIV/AIDS.

## Materials and methods

### The data

The study by Fairall and colleagues [1] included two datasets: Cohort 1 and Cohort 2 (see Table A and Table B in S1 File for the variable definitions for the two cohorts). The original authors provided us with primary outcomes for the two datasets in Stata format, along with the Stata code used to generate their results. The dataset for Cohort 1 includes patients aged 16 years and older with CD4 counts of ≤350 cells per μL who had not yet started ART [1]. The primary outcome for Cohort 1 was the time from enrollment to death. Secondary outcomes for Cohort 1 were measures of health status and indicators of quality of care. The data set for Cohort 2 includes patients who were adults, had already received ART for at least 6 months and were being treated at the time of enrollment. The primary outcome for Cohort 2 was the proportion of patients with undetectable viral load one year after enrollment. Secondary outcomes for Cohort 2 were measures of health status and indicators of quality of care. We generated findings based on these limited datasets, which included only the complete case data. Therefore, the results reported here may differ from those in the original study due to missing variables or discrepancies between the original and current datasets.

### Statistical methods

We first conducted a push button replication (PBR) study and then followed the statistical methods used in Fairall and others [1] to conduct the pure replication. We designed our pure replication to independently test the consistency of the original published results (Our replication paper is available at http://www.3ieimpact.org/media/filer_public/2017/11/29/rps13-hiv-treatment-south-africa.pdf). The study was restricted to the two primary outcomes analyses, due to limited access to the original data. The frequency (percentage) for categorical variables and the median (interquartile range [IQR]) for continuous variables were reported for baseline characteristics by cohort. In Cohort 1, time from enrollment to death was analyzed with Cox proportional hazards (PH) models and Huber-White robust adjustment of errors for intracluster correlation of outcomes. Comparisons of effect between intervention and control groups were conducted by reporting the number of deaths, person-months at risk and hazard of death per 100 person-months at risk with 95 percent confidence intervals (CI). All analyses were also stratified by baseline CD4 count groups (201–350 versus ≤200 cells per μL). In Cohort 2, binomial regression was used to estimate differences in proportions of patients with suppressed viral loads.

We next conduct a measurement and estimation analysis (MEA) to further evaluate the robustness of the original findings following the replication process described by Brown, Cameron and Wood [10]. We first checked the PH assumptions in the Cox PH model using the Schoenfeld residuals test and cumulative sums of martingale-based residuals methods [11] for the analysis of primary outcome in Cohort 1. If the PH assumption were violated for some predictors, then a stratified Cox model would be used to fit the data. To take the correlation of the responses in the same cluster into account, in the MEA we utilized two approaches: (1) the generalized estimating equation (GEE) approach [12] using the working correlation matrix; and (2) the frailty model [13, 14]. For the Cohort 2 study, to take the correlation of the responses (i.e. viral suppression one year after enrollment) in the same cluster into account, in the MEA we utilized two approaches: (1) the GEE approach [12]; and (2) the generalized linear mixed-effects model (GLMM) [15]. All the MEA analyses were conducted using R. This alternative coding language may have introduced slight differences from the original results.

## Results

### The push button replication result

The PBR results are reported in the Supporting Information. Table C in S1 File is the PBR result for Table 2 in the original paper of Fairall et al. [1], and Table D in S1 File is the PBR result for Table 4 in the original paper of Fairall et al. [1]. In Table C in S1 File, there are minor differences for the number of subjects in the subgroup analysis from the original results. We obtain n = 2,258 and 6,994 for the subgroups with baseline CD4 count 201–350 cells per μL and CD4 count <= 200 cells per μL, respectively, whereas the original results reported 2,283 and 6,969. The other replicated results are classified as comparable.

### The pure replication result

**Fig 1:**
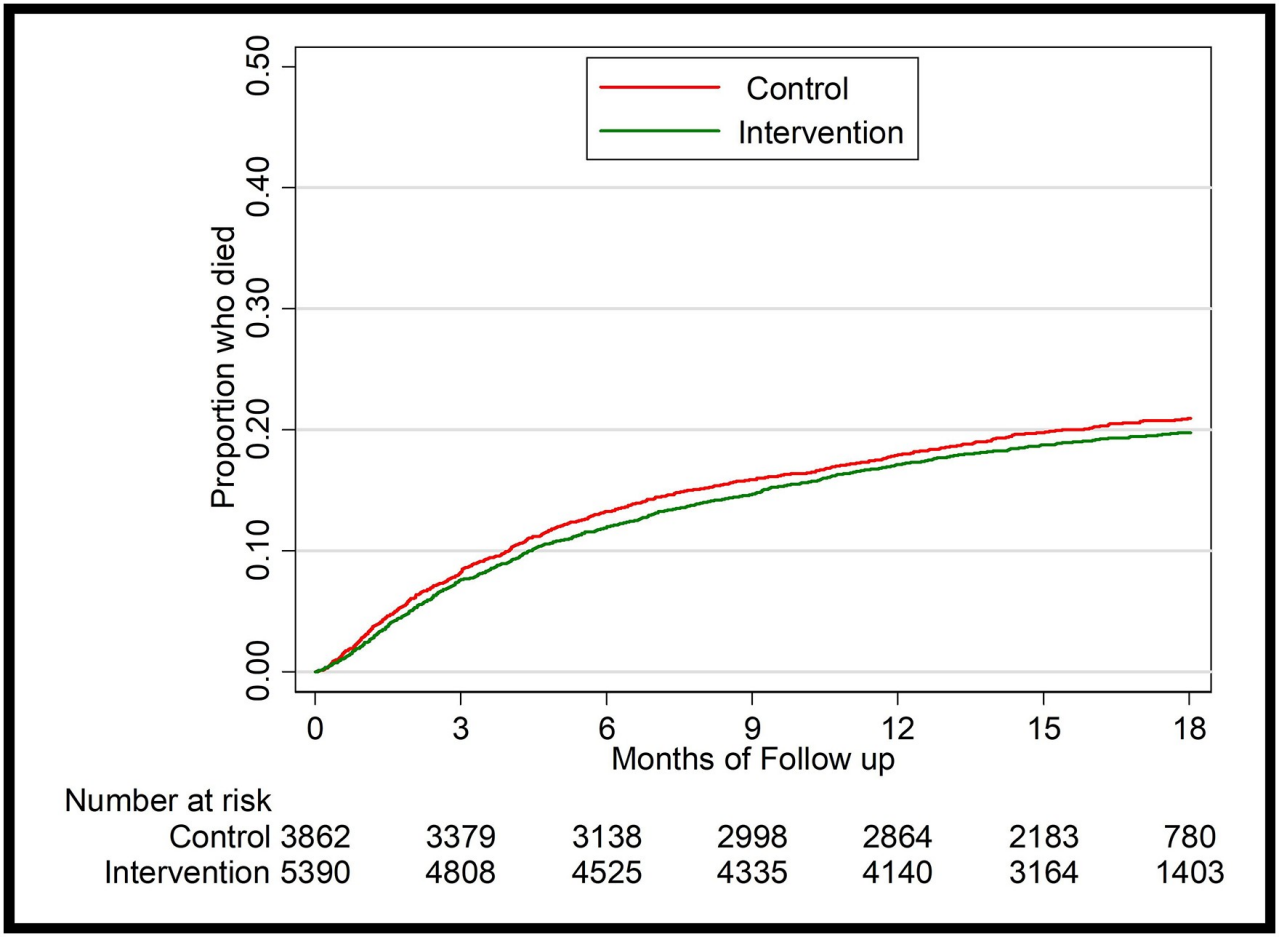
KM curves stratified by treatment groups in cohort 1.

**Fig 2:**
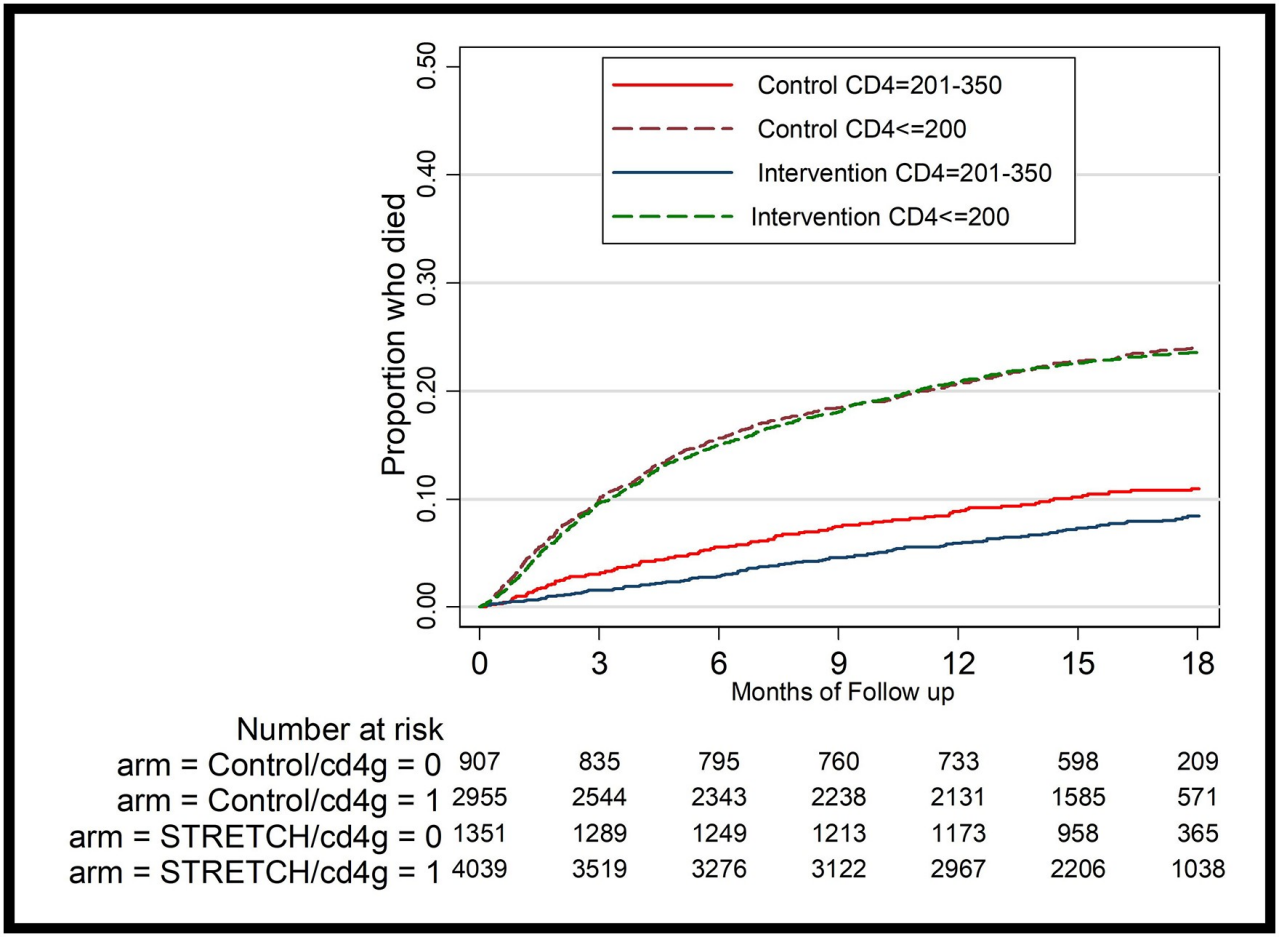
KM curve stratified by treatment and CD4 subgroups in cohort 1.

Table 1 reports the original and pure replication results for baseline characteristics by cohort, and Table 2 reports the original and pure replication results for the primary outcome in Cohort 1. We also reproduced the Kaplan-Meier failure curve of time to death (Fig 1) and for CD4 subgroups for Cohort 1 (Fig 2). Table 3 reports the original and pure replication results for the primary outcome in Cohort 2.

**Table 1:** Baseline characteristics by cohort to check the balance between the two treatment assignments: Original and replication results.

	Intervention group Original	Intervention group Replication	Control group Original	Control group Replication	P-value*
**Cohort 1**					
**Number of patients**	5,390	5,390	3,862	3,862	
**Women**	3,604 (67%)	3,604 (67%)	2,681 (69%)	2,681 (69%)	0.01
**Age (years)**	36 (30–43)	36 (30–43)	35 (29–42)	35 (29–42)	0.14
**National identity number recorded**	4,767 (88%)	4,767 (88%)	3,184 (82%)	3,184 (82%)	<0.01
**CD4 (cells per μL)**	141 (70–201)	141 (70–201)	137 (70–197)	137 (70–197)	0.28
**0–49**	934 (17%)	934 (17%)	678 (18%)	678 (18%)	
**50–99**	949 (18%)	949 (18%)	720 (19%)	720 (19%)	
**100–199**	2,141 (40%)	2,141 (40%)	1,547 (40%)	1,547 (40%)	
**200–350**	1,366 (25%)	1,366 (25%)	917 (24%)	917 (24%)	
**Cohort 2**					
**Number of patients**	3,029	3,029	3,202	3,202	
**Viral load <400 copies per mL**	2,378 (79%)	2,156 (71%)	2,507 (78%)	2,230 (70%)	0.19

Notes: Data are n (%), median (IQR), n/N (%).

* Test the difference between the intervention and control groups.

**Table 2:** Effect of the intervention on time from enrollment to death in Cohort 1: Original and pure replication results.

			Intervention group			Control group			Hazard ratio (95% CI)	Unadjusted/Crude p-value	Adjusted hazard ratio (95% CI)+	Adjusted p-value
		n	Number of deaths	Person-months at risk	Hazard of death per 100 person- months at risk (95% CI)*	Number of deaths	Person- months at risk	Hazard of death per 100 person-months at risk (95% CI)*				
Primary analysis	Original result	9,252	997	74,257	1.34 (1.26–1.43)	747	51,861	1.44 (1.34–1.55)	0.94 (0.76–1.15)	0.532	0.92 (0.76–1.12)	0.400
	Replication result	9,252	997	74,257	1.34 (1.26–1.43)	747	51,861	1.44 (1.34–1.55)	0.94 (0.76–1.15)	0.532	0.92 (0.76–1.12)	0.400
Subgroup analysis: baseline CD4 count 201–350 cells per μL	Original result	2,283	102	20,710	0.06 (0.03–0.10)	90	13,224	0.68 (0.55–0.84)	0.73 (0.54–1.00)§	0.052	0.70 (0.52–0.94)¶	0.019
	Replication result	2,258	102	20,710	0.49 (0.40–0.60)	90	13,224	0.68 (0.55–0.84)	0.73 (0.54–1.00)§	0.052	0.70 (0.52–0.94)¶	0.019
Subgroup analysis: baseline CD4 count ≤200 cells per μL	Original result	6,969	895	53,546	1.67 (1.56–1.78)	657	38,637	1.70 (1.57–1.83)	1.00 (0.80–1.24)	0.999	0.94 (0.77–1.15)	0.577
	Replication result	6,994	895	53,546	1.67 (1.56–1.78)	657	38,637	1.70 (1.57–1.83)	1.00 (0.80–1.24)	0.999	0.94 (0.77–1.15)	0.568

Note:

* Binomial exact confidence intervals.

^+^ Adjusted for patient’s age, sex, CD4 cell count at enrollment, and record of an identity number.

^§^ Interaction between group and CD4 cell count stratum p = 0.050.

^¶^ Adjusted for patient’s age, sex, and record of an identity number, interaction term between group and CD4 cell count stratum p = 0.049 for the original result and p = 0.047 for the replication result.

**Table 3:** Effect of the intervention on viral load in Cohort 2: Original and pure replication binomial regression results.

		Intervention group	Control group	Effect estimate*	P-value	Intracluster correlation coefficient
				Risk difference dstimate (95% CI)		
**Primary outcome**						
Suppressed viral load	Original result	2,156/3,029 (71.18%)	2,230/3,202 (70%)	1.1% (–2.3%-4.6%)	0.534	0.010
	Replication result	2,156/3,029 (71.18%)	2,230/3,202 (70%)	1.1% (–2.3%-4.6%)	0.534	0.010

Note:

* Regression models adjusted for randomization strata and intra-cluster correlation of outcomes.

Overall, our replication analysis conclusions are consistent with the original results, which indicate that time to death did not differ between the two groups when controlling for baseline characteristics (p = 0.400). In subgroup analysis with CD4 counts of 201–350 cells per μL, the intervention group patients had a 30 percent lower risk of death than those in the control group when controlling for baseline characteristics (p = 0.019). In subgroup analysis with CD4 counts of ≤200 cells per μL, time to death did not differ between the two groups when controlling for baseline characteristics (p = 0.568). Table 3 results indicate that viral suppression rates one year after enrollment did not differ between intervention and control patients.

### Measurement and estimation analysis results

Table 4 reports the MEA result for Cohort 1. For the primary analysis, all three methods (Original, GEE and Frailty) reached the same conclusions for both unadjusted and adjusted analyses.

**Table 4:** Effect of the intervention on time from enrollment to death in Cohort 1: Original and MEA results.

		Hazard ratio (95% CI)	Unadjusted/Crude P-value	Adjusted hazard ratio (95% CI)	Adjusted p-value
Primary analysis	Original result	0.94 (0.76–1.15)	0.532	0.92 (0.76–1.12)	0.401
	GEE analysis result	0.94 (0.76–1.15)	0.525	0.91 (0.75–1.11)	0.363
	Frailty model analysis result	0.91 (0.80–1.02)	0.194	0.89 (0.79–1.01)	0.077
Subgroup analysis: baseline CD4 count 201–350 cells per μL	Original result	0.73 (0.54–1.00)	0.052	0.70 (0.52–0.95)	0.020
	GEE analysis result	0.75 (0.60–0.95)	0.015	0.73 (0.56–0.94)	0.016
	Frailty model analysis result	0.76 (0.52–1.09)	0.130	0.72 (0.50–1.04)	0.079
Subgroup analysis: baseline CD4 count ≤200 cells per μL	Original result	1.00 (0.80–1.24)	0.999	0.94 (0.77–1.16)	0.577
	GEE analysis result	1.00 (0.80–1.24)	0.977	0.94 (0.77–1.13)	0.493
	Frailty model analysis result	0.97 (0.85–1.10)	0.620	0.92 (0.80–1.05)	0.190

In the unadjusted subgroup analysis with baseline CD4 count 201–350 cells per μL, the GEE analysis results showed that the hazard of death was significantly lower in the intervention group than in the control group (hazard ratio [HR] = 0.75, 95% CI: 0.60–0.95, p = 0.015). The original analysis (HR = 0.73, 95% CI: 0.54–1.00, p = 0.052) and frailty analysis (HR = 0.76, 95% CI: 0.52–1.09, p = 0.130) both showed non-significant results. The other conclusions were the same, although there were minor differences in the estimates. In the adjusted analysis, the GEE results (HR = 0.73, 95% CI: 0.56–0.94, p = 0.016) showed the same conclusion as in the original publication (HR = 0.70, 95% CI: 0.52–0.95, p = 0.020), although there were minor differences in the estimates. The frailty model analysis (HR = 0.72, 95% CI: 0.50–1.04, p = 0.079) showed a loss of significance from the original results.

In the subgroup analysis with baseline CD4 count ≤200 cells per μL, the GEE and frailty analyses both showed the same conclusion as in the original publication, although there were minor differences in the estimates.

We also applied the GEE and GLMMs to account for the cluster effects for the primary outcome in Cohort 2. We obtained the same conclusion as in the original result. See Table 5. For more details of the whole replication study, please refer our replication paper series at http://www.3ieimpact.org/media/filer_public/2017/11/29/rps13-hiv-treatment-south-africa.pdf.

**Table 5:** Effect of the intervention on viral load in Cohort 2: MEA results.

Methods	Odds ratio (95% CI)	P-value
Original result	1.1% (–2.3%-4.6%)*	0.534
GEE analysis result	1.12 (1.00–1.25)	0.054
GLMM result	1.08 (0.87–1.33)	0.484

Note:

* Risk difference and 95% CI.

## Discussion

We conducted the MEA by assessing the validity of model assumptions and proposed other advanced methods to assess the robustness of the conclusions reached by Fairall and colleagues in 2012.

Since the adjusted analyses control for potential confounders, we are more confident interpreting the adjusted analysis results than the unadjusted results. It may not be surprising that the frailty model or GLMM analysis showed a different conclusion from the original or GEE results, as the results from the two methods have different interpretations. The estimate from the GEE analysis has a marginal or population average interpretation, while the estimate from the frailty or GLMM analysis has a subject-specific inference. The GEE results are more meaningful to a policymaker, as they reflect population average inferences. The frailty or GLMM model results might be more meaningful for a patient.

Based on the GEE result for Cohort 1, shown in Table 4, the MEA generated the same conclusion as the original analysis: for the primary analysis and subgroup analysis with baseline CD4 count ≤200 cells per μL, time to death did not differ between intervention and control patients. In the subgroup analysis with baseline CD4 count 201–350 cells per μL, the intervention group patients had a 30 percent lower risk of death than those in the control group when controlling for baseline characteristics (Table 4). For Cohort 2 analysis, all methods yielded the same conclusions: rates of viral suppression one year after enrollment did not differ between the intervention and control groups.

This replication study focuses on the two primary outcomes in Cohorts 1 and 2. Though the original paper also analyzed secondary health outcomes and quality of care indicators, our replication study cannot evaluate findings for these outcomes due to limited data access. Another limitation of this study is that we cannot evaluate how the missing data will affect the conclusions. Fairall et al. [1] discussed the issue of incomplete data, “*We were missing data for weight and CD4 cell count in both cohorts*, *and for viral load after 12 months of ART in cohort 1”* [1], but they have not addressed the missing data issue. Due to limited data, we also cannot address this important issue.

## Conclusion

Although there are some minor differences between results of our analyses and the original paper, our replication study findings primarily validate the original findings. The minor differences may be due to discrepancies between the datasets or methods used in our analysis and in the original analysis. Overall, time to death did not differ between intervention and control patients, and rates of viral suppression one year after enrollment did not differ between the intervention and control groups. In subgroup analysis with CD4 counts of 201–350 cells per μL, the intervention group patients had a 30 percent lower risk of death than those in the control group when controlling for baseline characteristics. In subgroup analysis with CD4 counts of ≤200 cells per μL, time to death did not differ between the two groups. Although the intervention did not lead to improved well-being for all the main outcomes, it was proven safe to use, and it increased the pool of prescribers and their geographical range, which increased the quality of care of these patients [1].

The original authors have used a draft version of this replication study in a summary of all research on the intervention that they provided to the Government of South Africa’s National Department of Health [16]. They informed us that these replication results will be included in documentation around a further possible scale-up of the STRETCH intervention within South Africa in the near future. Our replication study enhances the confidence in implementation of task shifting of ART from doctors to trained nurses in developing countries similar to South Africa. Implementing the STRETCH program will benefit many HIV-positive patients in South Africa and other developing countries with similar circumstances without negatively influencing key health outcomes and while improving their quality of care. It can also relieve doctors from a heavy patient burden and enable them to focus on more severely ill patients. This is essential in South Africa and elsewhere where shortages of doctors restrict access to ART.

## Supporting information

S1 File Variable information and PBR results for cohorts 1 and 2. (DOCX)
