# Supplementary material for: STRETCHing HIV treatment: A replication study of task shifting in South Africa
Source: PLoS One. 2019 Apr 8;14(4):e0206677. doi: 10.1371/journal.pone.0206677 (PMC6453457; doi:10.1371/journal.pone.0206677)
Supplement: S1 File — (DOCX) [file pone.0206677.s001.docx]

Supporting Information for “STRETCHing HIV treatment: a replication study of task shifting in South Africa”

**Variable information and PBR results for cohorts 1 and 2.**

Table A: Variable definition for cohort 1-patients with CD4 ≤350 and not on ART at enrollment

| Variable name | Definition |
| --- | --- |
| survival time | Days from enrollment to death or censorship |
| death | 1: Died, 0: Did not die |
| arm | Stretch: intervention, Control: control |
| strata | Randomization strata |
| siteid | Randomization cluster |
| idcheck | National identification number recorded |
| sex | m: male, f: female |
| age at enrolment | age in years at enrollment |
| eligiblecd4value | CD4 count at enrollment (cells/microliter) |

Table B: Variable definition for cohort 2-patients on ART at enrolment

| Variable name | Definition |
| --- | --- |
| supprvl | Suppressed viral load (<400) |
| arm | Stretch: intervention, Control: control |
| strata | Randomization strata |
| siteid | Randomization cluster |

Table C: Effect of the intervention on time from enrollment to death in Cohort 1- A PBR of Fairall et al. Table 2

|  | **Intervention group** | | | **Control group** | |  | **Hazard ratio (95% CI)** | **P-value** | **Adjusted hazard ratio (95% CI)+** | **Adjusted p-value** |
| --- | --- | --- | --- | --- | --- | --- | --- | --- | --- | --- |
|  | Number of deaths | Person-months at risk | Hazard of death per 100 person-months at risk (95% CI)* | Number of deaths | Person-months at risk | Hazard of death per 100 person-months at risk (95% CI)* |  |  |  |  |
| Primary analysis (n=9252) |  |  |  |  |  |  | 0.94 (0.76-1.15) | 0.532 | 0.92 (0.76-1.12) | 0.400 |
| Subgroup analysis: baseline CD4 count 201-350 cells per µL (n=2258) |  |  |  |  |  |  | 0.73 (0.54-1.00)§ | 0.052 | 0.70 (0.52-0.94)¶ | 0.019 |
| Subgroup analysis: baseline CD4 count <=200 cells per µL (n=6994) |  |  |  |  |  |  | 1.00 (0.80-1.24) | 0.999 | 0.94 (0.77-1.15) | 0.568 |
| Note: +Adjusted for patient's age, sex, CD4 cell count at enrollment, and record of an identity number. §Interaction between group and CD4 cell count stratum p=0.050. ¶Adjusted for patient's age, sex, and record of an identity number, interaction term between group and CD4 cell count stratum p=0.047 | | | | | | | | | | |

Table D: Effect of the intervention on viral load in Cohort 2 – a PBR of Fairall and others (2012) Table 4

|  | **Intervention group** | **Control group** | **Effect estimate*** | | **P-value** | **Intracluster correlation coefficient** | **Regression model*** |
| --- | --- | --- | --- | --- | --- | --- | --- |
|  |  |  | Type | Estimate (95%CI) |  |  |  |
| **Primary outcome** |  |  |  |  |  |  |  |
| Suppressed viral load | 2156/3029 (71.18%) | 2230/3202 (70%) | Risk difference | 1.1% (-2.3%-4.6%) | 0.534 | 0.010 | Binomial |
| **Secondary outcomes**  Time to death  Program retention  New tuberculosis diagnosis  Received co-trimoxazole prophylaxis  Change in ART drugs during trial  Weight at follow-up (kg)  CD4 count at follow-up |  |  |  |  |  |  |  |
